# Case-control study of *GRIA1* and *GRIA3* gene variants in migraine

**DOI:** 10.1186/s10194-016-0592-2

**Published:** 2016-01-22

**Authors:** Jie Fang, Xingkai An, Shuai Chen, Zhenzhen Yu, Qilin Ma, Hongli Qu

**Affiliations:** Department of Neurology, The First Affiliated Hospital of Xiamen University, Xiamen, China; The First Clinical Medical College of Fujian Medical University, Fuzhou, China

**Keywords:** Migraine, GRIA1, GRIA3, Gene variant, Glutamate

## Abstract

**Background:**

As the most abundant excitatory neurotransmitter in the central nervous system, glutamate has been accepted to play a major role in the pathophysiology of migraine. The previous studies have reported the glutamate receptor ionotropic *GRIA1* and *GRIA3* genes variants associated with migraine. The project aims to investigate the polymorphisms in both genes for their association with migraine in the Chinese Han population.

**Methods:**

A Han-Chinese case-control population, including 331 unrelated female migraine patients and 330 matched controls, was studied. Variants in genes (*GRIA1* and *GRIA3*) were genotyped by Multiplex SNaPshot assay.

**Results:**

In the group of patients, the frequency of allele C was 84.1 % (557 C alleles) and allele T was 15.9 % (105 T alleles) for the *GRIA1* (rs2195450) in migraineurs, this was significantly as compared with the controls (*P* = .001, OR = 1.786, 95 % CI: 1.28–2.49). And an association was also seen in the migraine with aura (MA) subtype (*P* = .012, OR = 2.092, 95 % CI: 1.17–3.76) and migraine without aura (MO) subtype (*P* = .002, OR = 1.737, 95 % CI: 1.23–2.45). However, no evidence was found that *GRIA1* (rs548294) or *GRIA3* (rs3761555) is associated with migraine.

**Conclusion:**

Our data of this study confirmed the association of *GRIA1* (rs2195450) to female migraine (MA, MO) susceptibility in the Chinese Han population. The result provides evidence that the glutamatergic system is implicated in the pathophysiology of migraine.

## Background

Migraine, characterized by recurrent attacks of severe headache, is a complex debilitating neurovascular disorder accompanying with nausea, vomiting, photophobia and phonophobia, which can cause temporary incapacitation in the migraineur. It is divided into two common forms: migraine with/or without aura (MA and MO), and diagnosed according the ICHD-III(HIS 2013) [1]. Epidemiological studies have shown that migraine prevalence accounts for 12 % in Caucasian populations [2] and 9.3 % in mainland of China [3]. With severe influence on the patient’s physical and mental health, and even their normal social function, this disorder is seemed as a heavy burden for patents.

The exact causes and pathophysiological mechanisms that underlie migraine is elusive. It is generally considered to be associated with the genetic factors, about 50 % of affected individuals have a first-degree relative also suffering from migraine [4], with family and twin studies reporting heritability estimates from 34 to 57 % [5]. Current research suggests that the trigeminovascular system also plays an important role in migraine [6, 7], and neurotransmitters in this system include: serotonin, dopamine and glutamate implicated in migraine pathogenesis. The implication of glutamate in trigeminovascular activation, central sensitization and cortical spreading depression(CSD) [8–10] as well as the assessment of glutamate concentration in plasma, platelets and cerebrospinal fluid in clinical studies [11] argue for a significant role of the glutamatergic neurotransmission in the pathophysiology of migraine. Genetic association studies have mostly investigated variants in serotonin and dopamine receptor genes. Nevertheless, fewer studies have been done in relation to the genetics of the Glutamatergic system in migraine [12]. The study by Formicola et al. [13] who found a positive association with 2 SNPs in *GRIA1* and 1 SNP in the *GRIA3* in an Italian population of 250 migraineurs and 260 controls [13]. Recently, genome-wide association studies (GWAS) also support a correlation between migraine and variants in glutamatergic system genes [14–16].

However, involved in regulating neuronal glutamate signals lipoprotein receptor *LRP1* (rs11172113) gene variation, the gene mutation of *LRP1* has not been found in the Han population-based study [17]. Therefore, further research into the glutamate system is necessary to ascertain the gene variation in Han-population. Therefore, to determine if these variations in the *GRIA1* and *GRIA3* genes contribute to migraine susceptibility in an Chinese Han population case-control cohort, we report the findings of our association study of the 3 SNPs with a large sample involving 661 female individuals from southern Fujian province of China.

## Methods

### Subjects

Based on previous experience our study involves a relatively larger sample size. The study group comprised 331 Han-Chinese female migraine patients from outpatient of the Department of Neurology at the First Affiliated Hospital of Xiamen University during the period between September 2013 and May 2015. All of the migraine patients were diagnosed as having either migraine with (MA) or migraine without (MO) aura, by two headache specialists after neurological examination, direct interview, computed tomography (CT), or magnetic resonance imaging (MRI), according to the diagnostic criteria set by the International Headache Society (international classification of headache disorders,3rd edition, 2013) [1]. The control group comprised 330 non-headache healthy female volunteers, who were recruited from the same regional background (southern Fujian province), and matched for age and gender with the study group. Familial Hemiplegic Migraine, tumor, depression and other comorbid psychiatric disorders were excluded from the study. The Ethics Committee of the First Affiliated Hospital of Xiamen University approved this study, and informed consent was obtained from all participants and volunteers.

### Genetic analysis

Genomic DNA from each participant was extracted from peripheral blood with ethylene diamine tetraacetic acid (EDTA) anticoagulant. Genomic DNA was prepared by a standard extraction method using the QiaAmp DNA Mini Kit (Qiagen, Hilden, Germany) and the DNA samples were stored at −20 °C before being used. The three SNPs were selected for this study rs548294 and rs2195450 in *GRIA1*, rs3761555 in *GRIA3* were genotyped using Multiplex SNaPshot technique (Applied Biosystems by Life Technologies, Foster City, CA, USA). Table 1 shows the primers of PCR and SNaPshot reaction. PCR reactions containing 25 μl final volume mixture (50 mM MgCl_2_, 10 mM dNTP, 1 μM primers, 5 units Platinum Taq DNA polymerase). The PCR conditions used were: 95 °C denaturation for 2 min, amplification reaction for 33 cycles (95 °C for 20s, 55 °C for 20s, 72 °C for 40s), with a final extension step of 72 °C for 5 min. The PCR products were examined on a 1.5 % agarose gel whose fragments indicate successful experiment. SNaPshot reaction contains 5 μl final volume mixture (Reaction mix 2.5 μl, PCR products 1.5 μl, Probe Mix 1.0 μl). SNaPshot reaction procedure is as following: amplification reaction for 25 cycles (96 °C for 10s, 50 °C for 5 s, 60 °C for 30s), with a final extension step of 60 °C for 1 min. DNA sequencing: a 10 μl final volume mixture (SNaPshot purified product 1 μl, Formamide Deionized 8.5 μl, GeneScan-120 LIZ Size Standard 0.5 μl). 95 °C denaturation 5 min, and sequenced using an ABI PRISM 3730 DNA Sequencer (Applied Biosystems by Life Technologies, Foster City, CA, USA). All sequence analyses were performed using it’s own DNA Sequencing Analysis software, GeneMapper4.0.

**Table 1:** PCR and SNaPshot Probe Primer Sequences

Polymorphisms	Primers	Product size	Sequence 5′ → 3′
GRIA1	Forward	297bp	CACCCCAAGTTTTCACTGCT
rs548294	Reverse	297bp	GAACCTAAAAGATCCCCAGGT
	Probe	63bp	37Ts+CTTAACTCTTAGGCTCTAATA
GRIA1	Forward	259bp	GCTGGAGGAGTCCAGAACAG
rs2195450	Reverse	259bp	TGCTTGGTAGATGGTGCTTG
	Probe	70bp	45Ts+CCCCTCACTCCTTCTTCT
GRIA3	Forward	373bp	TGCCAGTGTAAAGCGAACTG
rs3761555	Reverse	373bp	GGGGATGACCAACCTTACCT
	Probe	56bp	35Ts+TTCAAAATGGAGACAAAAGAT

### Statistical analysis

To assess the deviation of each polymorphism from the Hardy-Weinberg equilibrium (HWE), the public statistics web tool (http://ihg.gsf.de/cgi-bin/hw/hwa1.pl) was employed. All statistical analyses were performed using the statistical package SPSS version 20.0 for Windows (SPSS, Inc., Chicago, IL, USA). The Chi-squared test or t-test was used to compare age and sex among the groups, allele and genotype frequencies were compared using the Chi-squared test. P values were adjusted for multiple testing using the Bonferroni correction, therefore, we used *P* < .05/3 ≈ .017 as a threshold for significance.

## Results

### General information

This study involved a total of 331 female migraine patients and 330 female controls. The mean age of the migraine patients was 35.0 ± 7.6 years, and the controls were 34.4 ± 7.2 years. The migraine patients and the controls were matched with respect to age and gender (*P* = .255/1.00). There are 47 MA and 284 MO included in the migraine patients group. Two SNPs (rs548294 and rs2195450) in the *GRIA1* gene and one SNP (rs3761555) in the *GRIA3* gene that have previously demonstrated positive association to migraine in the study by Formicola et al. were selected and tested in our study [13]. All the genotypic distributions analyzed in patients and controls were in accordance with the Hardy-Weinberg equilibrium (*P* > .05).

### *GRIA1* polymorphisms

No significant difference in genotypic and allelic distribution was observed in the polymorphisms of rs548294 between migraine cases and controls. And also, difference was not observed for any of the migraine subtypes (MA, MO) tested (Tables 2 and 3).

**Table 2:** Genotype frequencies of gene polymorphism in Chinese population

Gene	SNP	Group		Genotype n(%)		P
*GRIA1*	rs548294		GG	GA	AA	
		Migraine	159(48.0)	130(39.3)	42(12.7)	.642
		MA	28(59.6)	16(34.0)	3(6.4)	.280
		MO	131(46.1)	114(40.1)	39(13.7)	.494
		Control	157(47.6)	138(41.8)	35(10.6)	
	rs2195450		CC	CT	TT	
		Migraine	238(71.9)	81(24.5)	12(3.6)	.004*
		MA	31(66.0)	15(31.9)	1(2.1)	.027
		MO	207(72.9)	66(23.2)	11(3.9)	.011**
		Control	272(82.4)	53(16.1)	5(1.5)	
*GRIA3*	rs3761555		GG	GA	AA	
		Migraine	135(40.8)	154(46.5)	42(12.7)	.564
		MA	21(44.7)	19(40.4)	7(14.9)	.864
		MO	114(40.1)	135(47.5)	35(12.3)	.470
		Control	134(40.6)	145(43.9)	51(15.5)	

*Abbreviations*: *MA* migraine with aura, *MO* migraine without aura, *SNP* single nucleotide polymorphisms

*All female migraine patients compared with controls by genotype: *P* < .017

**MO compared with controls by genotype: *P* < .017

**Table 3:** Allele frequencies of gene polymorphism in Chinese population

Gene	SNP	Group	Allele n(%)		P
GRIA1	rs548294		G	A	
		Migraine	448(67.7)	214(32.3)	.752
		MA	72(76.6)	22(23.4)	.110
		MO	376(66.2)	192(33.8)	.394
		Control	452(68.5)	208(31.5)	
	rs2195450		C	T	
		Migraine	557(84.1)	105(15.9)	.001*
		MA	77(81.9)	17(18.1)	.012**
		MO	480(84.5)	88(15.5)	.002***
		Control	597(90.5)	63(9.5)	
GRIA3	rs3761555		G	A	
		Migraine	424(64.0)	238(36.0)	.579
		MA	61(64.9)	33(35.1)	.663
		MO	363(63.9)	205(36.1)	.629
		Control	413(62.5)	247(37.4)	

*Abbreviations*: *MA* migraine with aura, *MO* migraine without aura, *SNP* single nucleotide polymorphisms, *CI* confidence interval

*All female migraine patients compared with controls by allele: odds ratio =1.786, 95 % CI: 1.28–2.49, *P* < .017

**MA compared with controls by allele: odds ratio =2.092, 95 % CI: 1.17–3.76, *P* < .017

***MO compared with controls by allele: odds ratio =1.737, 95 % CI: 1.23–2.45, *P* < .017

The frequencies of the genotypes of the SNP rs2195450 marker showed significant difference between migraine cases and controls (*P* = .004), and also a positive association with the allelic distribution was observed with an overrepresentation of the T allele (Tables 2 and 3). In the group of patients, the frequency of allele C was 84.1 % (557 C alleles) and allele T was 15.9 % (105 T alleles) for the *GRIA1* (rs2195450) in migraineurs, this was significantly as compared with the controls (*P* = .001, OR = 1.786, 95 % CI: 1.28–2.49). And an association was also seen in the MA subtype (*P* = .012, OR = 2.092, 95 % CI: 1.17–3.76) and MO subtype (*P* = .002, OR = 1.737, 95 % CI: 1.23–2.45).

### *GRIA3* polymorphisms

No significant difference in genotypic and allelic distribution was observed in the polymorphisms of rs3761555 between migraine cases and controls. And also, difference was not observed for any of the tow migraine subtypes tested (Tables 2 and 3).

## Discussion

Glutamate is the most abundant excitatory neurotransmitter in the brain,glutamate and the receptors are intimately involved in trigeminovascular activation, central sensitization and cortical spreading depression. Alterations in the function or expression of components of this system may be involved in migraine susceptibility [12]. Biochemical, preclinical experiments and pharmacological studies also support involvement of glutamate in migraine [18–20].

In addition, the previous studies have reported the significant role of glutamatergic gene variants played in the pathophysiology of migraine. GWAS supported that genetic variants (rs1835740 at 8q22.1 and rs11172113 in *LRP1*)associated with migraine [16, 21, 22], and both of these tow genetic variants were involved in the regulation of glutamatergic system [23]. Another association analysis on Italian population have reported two genes that code for subunits of the ionotropic AMPA receptors *GRIA1* and *GRIA3* have been implicated in migraine [13]. Although a number of studies have reported associations between particular glutamatergic gene variants and migraine susceptibility, replication studies to confirm previous findings are generally lacking [24]. For instance, as for the *LRP1* gene variation, our previous results [17] and another research-based Chinese Han population [25] both failed to replicate the association between rs11172113 and migraine. The present study is the first reported case-control association study to independently replicate the variations in the *GRIA1* and *GRIA3* genes in Chinese Han population.

In our present study, a statistically significant finding is that the *GRIA1* rs2195450 variant is a potential genetic risk factor for female migraine in the Chinese Han population from the southern Fujian province of China. In the subgroup analysis, the variant indicates association with the MA, MO subtype. This result was not as same as the previous reports. In 2010, Formicola and colleagues first reported the rs2195450 SNP of *GRIA1* gene was associated with the MA subgroup of migraineurs in Italian Caucasian population [13]. However, the SNP rs2195450 was in Hardy-Weinberg disequilibrium (DHW) both in controls and in patients, suggesting the possibility in the study of inappropriate population stratification and selection or other confounding factors.

Subsequently, two independent studies in Australia [26] and Italy Caucasian population [27] have both failed to identify the association of rs2195450 with migraine, either MA or MO. But it should be noted that the study in Australia population, the rs2195450 SNP was in DHW in the control group, and the study in Italy Caucasian population only included 186 patients. Compared with previous studies, our study involves a relatively larger sample size. Moreover, the rs2195450 SNP was found in HWE both in controls and in patients. Nonetheless, an even larger scale case-control study in populations of different origin should be performed to evaluate the association of the *GRIA1* rs2195450 variant to migraine susceptibility.

However, no association of the other SNP (rs548294) of *GRIA1* gene with migraine was found in our study, even in the subtype’s analysis. Although the previous results manifests that rs548294 SNP was significantly associated with migraine (MO and Female) in Italian Caucasian population [13]. But subsequent replication studies of this variant carried out in Italian Caucasian population failed to identify the association of rs548294 with migraine. Furthermore, when the two Italian Caucasian population studies were combined for pooled analysis, none of the genetic models testes provided a significant association between rs548294 polymorphism and MO risk [27]. In addition, another study in Australia population reported no evidence of association between rs548294 and risk of migraine [26]. Existing studies indicates that this variant may be not a risk factor for migraine.

Similarly, the rs3761555 SNP of *GRIA3* gene was also found no association with MA or MO in our study. This result was opposite to the previous two findings. The two former studies have showed the same results that rs3761555 SNP was significantly associated with migraine (MA and Female) [26, 27]. These controversial results may be caused by ethnic differences, environmental effects, and limited sample size.

## Conclusions

This study is the first report of rs2195450 SNP in *GRIA1* gene associated with female migraine (MA, MO) in Chinese Han population. The result also provides evidence that the glutamatergic system, particularly the *GRIA1* gene, is implicated in the pathophysiology of migraine. Further research into the glutamatergic system is necessary to ascertain the mechanism in the aetiology of migraine.
